# Optimisation of Lapping Process Parameters for Single-Crystal 4H–SiC Using Orthogonal Experiments and Grey Relational Analysis

**DOI:** 10.3390/mi12080910

**Published:** 2021-07-30

**Authors:** Jiayun Deng, Qiusheng Yan, Jiabin Lu, Qiang Xiong, Jisheng Pan

**Affiliations:** School of Mechanical and Electrical Engineering, Guangdong University of Technology, Guangzhou 510006, China; dengjiayun@mail2.gdut.edu.cn (J.D.); xiongqiang@gdut.edu.cn (Q.X.); panjisheng@gdut.edu.cn (J.P.)

**Keywords:** orthogonal experiment, grey relational analysis, lapping process parameter optimisation, single-crystal 4H–SiC

## Abstract

Lapping is one of the standard essential methods to realise the global planarization of SiC and other semiconductor substrates. It is necessary to deeply study the mechanism to obtain SiC lapping process parameters with a strong comprehensive lapping performance (i.e., high material removal rate (MRR_m_), small surface roughness (Ra), and low total thickness variation (TTV)). The effects of the lapping process parameters and their interactions on lapping performance for SiC were investigated using orthogonal experiments; the effects on the MRR_m_, Ra, TTV, and optimal parameters under the conditions of a single evaluation index were investigated using intuitive analysis (range analysis, variance analysis, and effect curve analysis). The entropy value method and grey relational analysis were used to transform the multi-evaluation-index optimisation into a single-index optimisation about the grey relational grade (GRG) and to comprehensively evaluate the lapping performance of each process parameter. The results showed that the lapping plate types, abrasive size, and their interaction effect had the most significant effects on MRR_m_ and Ra, with a contribution of over 85%. The interaction between the lapping plate types and abrasive size was also found to have the most significant effect on TTV, with a contribution of up to 51.07%. As the lapping plate’s hardness and abrasive size increased, the MRR_m_ and Ra also gradually increased. As the lapping normal-pressure increased, MRR_m_ increased, Ra gradually decreased, and TTV first decreased and then increased. MRR_m_, Ra, and TTV first increased and then decreased with increasing abrasive concentration. Compared to the optimisation results obtained by intuitive analysis, the process parameter optimised by the grey relational analysis resulted in a smooth surface with an MRR_m_ of 90.2 μm/h, an Ra of 0.769 nm, and a TTV of 3 μm, with a significant improvement in the comprehensive lapping performance. This study reveals that a combination of orthogonal experiments and grey relational analysis can provide new ideas for optimising the process parameters of SiC.

## 1. Introduction

**Figure 1:**
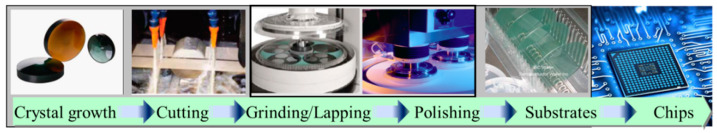
Processing flow of SiC substrates.

As one of the most promising third-generation semiconductor materials, single-crystal 4H–SiC is of great interest due to its excellent electrical, mechanical, and chemical properties, such as forbidden bandwidth, high breakdown electric field, high electron mobility, high breakdown strength, and high thermal conductivity. Furthermore, it is suitable for preparing electronic devices under extreme environmental conditions, such as high voltage, high frequency, high power, and high temperature [1,2]. To prepare electronic devices based on a 4H–SiC substrate, a damage-free, ultra-smooth atomic-level surface with a high surface flatness (total thickness variation (TTV); TTV ≤ 15 μm) and low surface roughness (Ra; must be maintained at values less than 0.3 and 0.5 nm for the C- and Si-faces, respectively) is required [3]. A single-crystal 4H–SiC for an electronic device’s preparation requires a series of processing processes, such as cutting, grinding/lapping, and polishing (as shown in Figure 1). The processing technology determines the surface quality of the SiC and directly affects the device’s preparation level and performance. However, due to its high hardness, high brittleness, and high stable chemical properties, the high-quality and high-efficiency surface grinding/lapping/polishing processing is challenging [4,5].

Grinding/lapping is a critical planarization process approach to obtain a high-quality single-crystal 4H–SiC substrate surface, mainly using high-hardness wheels/abrasives to efficiently remove cut marks and rough peaks on the sliced SiC substrate’s surface to reduce surface roughness and improve surface flatness for polishing [6]. However, during the grinding process, the grinding wheel and the substrate have a two-body friction movement that can lead to a strong TTV [7,8], though with relatively greater subsurface damage. In order to reduce the polishing time while improving the TTV and minimizing sub-surface damage, lapping (which mainly comprises three-body friction movement and less two-body friction movement) can be used. Luo et al. [9] used fixed and semi-fixed diamond abrasive tools to conduct comparative lapping processing experiments on SiC substrates. The MRR_m_ values were reduced from 130.5 to 2.7 nm/min, and the processed Ra was reduced from 90 to 35.5 and 5.1 nm, respectively; this showed that lapping processes can quickly reduce surface roughness and improve surface quality. Li et al. [10] investigated the effect of abrasive properties on the lapping performance of single-crystal 6H–SiC, and their results showed that an abrasive concentration of 7.69 wt% could result in a strong lapping effect, and an abrasive mixed with diamond and boron carbide in a certain ratio could also obtain a strong surface quality and high lapping efficiency. Zhao et al. [11] experimentally studied the lapping process of single-crystal SiC, and a surface with a TTV of less than 5 μm and an Ra of 0.12 μm was obtained. Su et al. [12] and Liang et al. [13] also studied the effect of the lapping process parameters on the lapping processing quality of single-crystal SiC, and their results showed that lapping normal-pressure and abrasive size had the greatest effect on MRR_m_. Tam et al. [14] and Hu et al. [15] found that the higher the abrasive hardness, the larger the abrasive size, and the higher the lapping plate hardness, the higher the MRR_m_ and Ra values after processing. Increasing the abrasive concentration led to the MRR_m_ first increasing and then decreasing and the Ra gradually increasing, and increasing the lapping normal-pressure and the lapping rotation speed led to the MRR_m_ also gradually increasing. However, though the Ra increased as the lapping normal-pressure increased, the Ra first increased and then decreased with the increase in the lapping rotation speed. These studies showed that lapping processing is a complex process involving multiple process parameters and their interactions. Most reports have only used single-factor experiments or orthogonal experiments without considering the interactions between various parameters to study the effects of each process parameter on the processing quality of SiC, which makes it difficult to comprehensively reveal the mechanisms of the process parameters and their interactions on the lapping processing of SiC. In addition, in current evaluations of the processing technology, a single evaluation index (MRR_m_ or Ra), instead of both or more, is usually used to evaluate the processing process.

An orthogonal design can use a small number of experiments to comprehensively analyse the effects of the process parameters and their interactions on the results, distinguish the significant and insignificant parameters, and obtain the best process parameter combination. The grey relational analysis method can determine the relational grade between the parameters and experimental results based on the similarity or dissimilarity of the development trend among the process parameters, which is significantly better than the orthogonal design in solving multi-evaluation-index optimisation problems and has been successfully applied in many engineering fields, such as laser machining, mechanism design, and ultra-precision milling and grinding. Senthilkumar et al. [16] used orthogonal experimental and grey relational analysis to optimise the problem of transformer oil blended with natural ester oils. Kursuncu et al. [17] also used orthogonal experimental and grey relational analysis to optimise the cutting parameters problem in the minimum quantity of the lubrication-assisted face milling of AISI O2 steel; their research showed that the combination of orthogonal experiments and grey relational analysis is better than either method alone in solving the optimisation problems among multiple parameters and multiple evaluation indexes.

In this study, an orthogonal experiment was used to study the effects of lapping plate type, lapping normal-pressure, abrasive size, abrasive concentration, and their interactions on the MRR_m_, Ra, and TTV to optimise the lapping process parameters of single-crystal SiC; the entropy value method was also used to assign weights for each evaluation index, and grey relational analysis was used to transform the optimisation problem of multiple evaluation indexes into a single-index optimisation problem regarding the grey relational grade (GRG), to obtain the lapping process parameters with a strong, comprehensive performance and provide new ideas for the lapping processing and process parameter optimisation of SiC.

## 2. Materials and Experiments

### 2.1. Experimental Principles and Device

**Figure 2:**
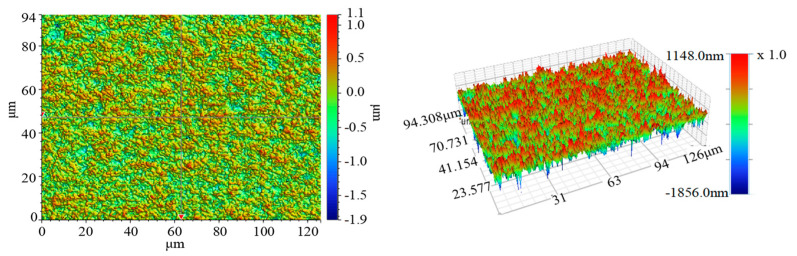
Surface morphology of the SiC substrate before lapping.

The experiments were conducted on single-crystal 4H–SiC substrates (C-face, a diameter size of 20.8 mm, research-grade, and made by TankeBlue Semiconductor Co. Ltd., Beijing, China), which were cut by a solid diamond abrasive wire saw, with rough surfaces and many rough peaks. The initial surface roughness, Ra, was about 180 nm, and the surface morphology is shown in Figure 2.

If it is directly processed by chemical mechanical polishing, the Ra of an SiC substrate is about 40–50 nm after polishing for 1 h, and a large number of rough peaks still remain on the surface. It is difficult to obtain a surface with global nano-level roughness [18]. Therefore, it was necessary to roughly lap-process the 4H–SiC substrate to reduce the Ra to less than 10 nm and then continually process it with chemical mechanical polishing to achieve a global sub-nanometre surface roughness that met the application requirements.

**Figure 3:**
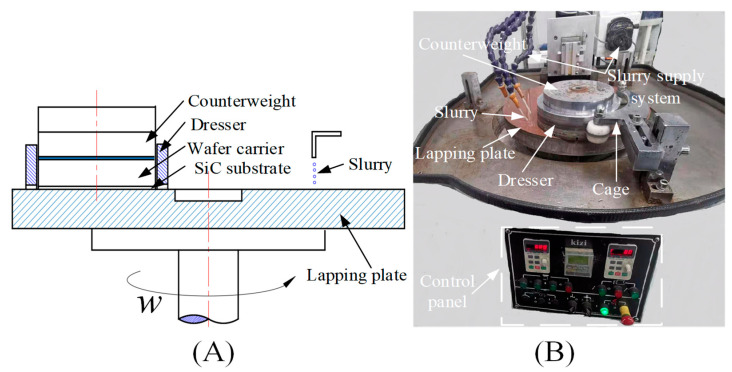
The principle (**A**) and device (**B**) for the lapping experiment of the SiC substrate.

The lapping experiment device for the SiC substrate is shown in Figure 3. The substrate was pasted onto a circular ceramic plate wafer carrier by paraffin wax, and the counterweight was placed directly above the wafer carrier. The lapping normal-pressure was changed by adjusting the counterweight, the wafer carrier coated the dresser ring, and the pulley of the cage mechanism was tangent to the dresser ring; when the lapping plate rotated, the dresser ring and the wafer carrier automatically rotated under the friction action between the holding mechanism and the lapping plate and the lapping slurry was dripped into the working area by the slurry supply system to achieve the lapping of the substrates.

### 2.2. Experimental Design and Methods

#### 2.2.1. Theoretical Basis for the Experimental Design

The classical Preston equation based on continuous material removal by abrasives is widely used to describe the MRR in high-volume manufacturing processes. It is believed that the MRR is linearly related to the lapping normal-pressure (*P*) and relative velocity (*V*), as shown in Equation (1). When abrasives are pressed into the substrate surface to a certain depth under the action of the lapping normal-pressure, a plough groove is drawn on the substrate surface under the drive of the relative speed. A large number of abrasives participate in the lapping to achieve the final removal in the continuous lapping process.

(1) MRR=K∗P∗V

where *K* is the Preston constant, which represents the effect of the remaining process parameters on MRR.

According to the research of Evansa et al. [19], besides lapping normal-pressure and relative velocity affecting MRR in lapping processes, the other process parameters, such as lapping plate hardness, abrasive type, abrasive size, and the variation in the actual contact area between the substrate and the lapping plate, can also affect MRR.

**Figure 4:**
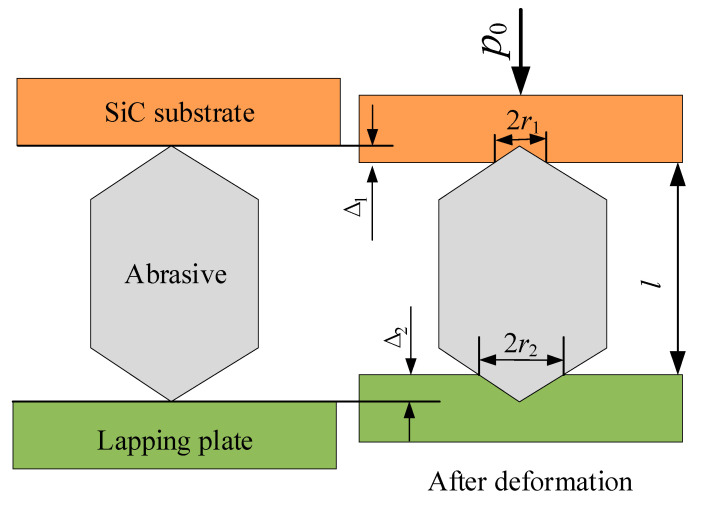
The contact model among an abrasive, an SiC substrate, and a lapping plate.

Hu et al. [20] established a contact model among an abrasive, an SiC substrate, and a lapping plate, as shown in Figure 4. According to the deformation amount at the contact area (Equation (2)), the material removal rate *V*_r_ of a single abrasive can be expressed as Equation (3), and if the abrasives are evenly distributed on the surface of the SiC substrate and the lapping plate, the material removal mathematical model of MRR_m_ for the mass of the SiC can be expressed as Equation (4).

(2) $$\Delta =\Delta_{1}+\Delta_{2}=\frac{p_{0}d_{s}\left(1/H_{s}+1/H_{p}\right)}{2\pi {\left(\;3r/4\rho_{p}*p_{0}\left[\left(1-v_{p}^{2}\right)E_{s}+\left(1-v_{s}^{2}\right)E_{p}\right]/E_{s}E_{p}\right)}^{\frac{2}{3}}\rho_{p}}$$

(3) $$V_{r}=\Delta_{1}r_{1}v=\frac{4\sqrt{2p_{0}v/\rho_{p}}{\;d}_{a}^{2}E_{p}}{9r{\left(\pi H_{s}\right)}^{\frac{3}{2}}}$$

(4) $${MRR}_{m}=\rho_{s}{NV}_{r}$$

where *N* is the adequate number of active abrasives in the lapping process according to the abrasive size distribution rules and the contact form among the abrasive, SiC substrate, and lapping plate during the lapping process; *N* can be expressed as Equation (5).

(5) $$N=\frac{3{D\rho }_{1}{C\rho }_{p}d_{s}{}^{2}A_{avg}h}{2\rho_{a}d_{a}^{3}}*\left(\Phi \left[3-\frac{0.25*{\left(4/3\right)}^{\frac{2}{3}}\left(d_{avg}+3\sigma_{a}\right)\left(2/H_{s}+1/H_{p}\right)E_{p}^{\frac{2}{3}}p_{0}^{\frac{1}{3}}}{\pi \sigma_{a}\rho_{p}^{\frac{1}{3}}{\left(3r/4\right)}^{\frac{2}{3}}}\right]\right)$$

In Figure 4 and related equations, *p*_0_ is the lapping normal-pressure applied to the substrate; Δ, Δ_1_, Δ_2_, *r*_1_, *r*_2_, and *l* are the total deformation amount among the SiC substrate and the lapping plate, the total deformation amount among the substrate and the abrasive, the total deformation amount among the abrasive and the lapping plate, the contact region radius of the substrate and the abrasive, the contact region radius of the abrasive and the lapping plate, and the distance between the substrate surface and the lapping plate surface, respectively; *H_s_*, *E_s_*, *v_s_*, *d_s_*, and *ρ_s_* are the hardness, Young’s modulus, Poisson’s ratio, diameter, and density of the SiC substrate, respectively; *H_p_*, *E_p_*, and *v_p_* are the hardness, Young’s modulus, and Poisson’s ratio of the lapping plate, respectively; *r*, *ρ_p_*, and *h* are the average curvature radius, distribution density, and average deformation height of a single projection of the lapping plate, respectively; *v* is the relative velocity of the substrate and lapping plate; *d_a_*, *d_avg_*, *σ_a_*, and *ρ_a_* are the size, average size, standard deviation, and density of the effective active abrasive in the lapping area, respectively; *A_avg_* is the average contact area between the substrate and the projection of the lapping plate; and *ρ*_1_, *C*, and *D* are the density, concentration, and dilution ratio of the abrasive before dilution, respectively.

By substituting Equations (3) and (5) into Equation (4), the mathematical model for the MRR_m_ of SiC during the lapping process can be obtained, as shown in Equation (6).

(6) $${MRR}_{m}=\frac{2\rho_{s}\rho_{1}\rho_{p}A_{avg}{hCDd}_{s}{}^{2}\sqrt{2p_{0}v/\rho_{p}}{\;E}_{p}}{3\rho_{a}d_{a}r{\left(\pi H_{s}\right)}^{\frac{3}{2}}}*\left(\Phi \left[3-\frac{0.25*{\left(4/3\right)}^{\frac{2}{3}}\left(d_{avg}+3\sigma_{a}\right)\left(2/H_{s}+1/H_{p}\right)E_{p}^{\frac{2}{3}}p_{0}^{\frac{1}{3}}}{\pi \sigma_{a}\rho_{p}^{\frac{1}{3}}{\left(3r/4\right)}^{\frac{2}{3}}}\right]\right)$$

From Equation (6), it can be seen that the MRR_m_ of SiC during the lapping process is mainly affected by the properties of the lapping plate (determined by *H_p_*, *E_p_*, *r*, *ρ_p_*, and *h*), the properties of the SiC substrate (determined by *H_s_*, *d_s_*, and *ρ_s_*), the properties of the lapping slurry (determined by the *d_a_*, *d_avg_*, *σ_a_*, *ρ_a_*, *ρ*_1_, *C*, and *D*), the lapping normal-pressure (*p*_0_), the relative rotational speed (*v*), the contact state between the substrate and the lapping plate (determined by *A_avg_*), and the interactions between the process parameters.

#### 2.2.2. Experimental Design

Following Equation (6), process parameters such as lapping plate type (A), lapping normal-pressure (B), abrasive size (C), and abrasive concentration (D), as well as the effects of their interactions, were selected to study the effects of various parameters on the processing quality and efficiency of single-crystal SiC during the lapping process. A four-factor, a three-level orthogonal experiment was designed with MRR_m_, Ra, and TTV after lapping as evaluation indexes, and the specific experimental parameters and levels are shown in Table 1; the L_27_(3^13^) orthogonal array was chosen to design this experiment, and the orthogonal array table header is shown in Table 2. Other experimental conditions were as follows: the working slurries were diamond abrasive lapping slurries of different concentrations and sizes with a flow rate, lapping time, and lapping plate speed of 15 mL/min, 30 min, and 40 r/min, respectively. Furthermore, each experiment was conducted 3 times.

### 2.3. Experimental Characterisations

#### 2.3.1. Material Removal Rate (MRR_m_)

A high-resolution electronic balance (with an accuracy of 0.1 mg) was used to measure the SiC weight loss before and after lapping. The MRR_m_ (μm/h) was calculated using Equation (7) to evaluate the processing efficiency with the average value, and the standard deviation was the MRR_m_ variation range.

(7) $$MRR_{m}=\frac{4\Delta m*10^{4}}{\rho_{s}\pi d_{s}{}^{2}t}$$

where Δ*m* (g), *ρ_s_* (g/cm^3^), *d_s_* (mm), and *t* (h) are the weight loss with lapping, density, diameter, and lapping time of the SiC substrate under lapping, respectively.

#### 2.3.2. Surface Roughness (Ra)

A white light interferometer (BRUKER Contour GT-X) was used to detect the surface roughness, Ra, of the centre point and four symmetrical points on the circumference 4 mm from the substrate’s edge (two of the points were located on a diameter at an angle of 30° counter clockwise to the perpendicular bisector of the substrate’s primary reference plane, and the other two points were located on another diameter perpendicular to this diameter), the average value of five points was used as the Ra evaluation index for the surface quality before and after lapping, and the standard deviation was the Ra variation range.

#### 2.3.3. Total Thickness Variation (TTV)

A micrometre was used to detect the substrate thickness of the centre point and four symmetrically located points on the d_s_/10 circumference from the substrate edge (two of the points were located on a diameter at an angle of 30° counter clockwise to the perpendicular bisector of the substrate’s primary reference plane, and the other two points were located on another diameter perpendicular to this diameter). The difference between the maximum and minimum of the five thickness values was used as the TTV to characterise the substrate surface flatness before and after lapping.

## 3. Experimental Results and Analysis

The experimental device is shown in Figure 3, and the parameters shown in Table 1 and Table 2 were used to carry out the lapping process parameter experiments. The experimental results are shown in Table 3. The range analysis, variance analysis, effect curve analysis, and grey relational analysis were carried out based on the experimental results.

### 3.1. Range Analysis and Variance Analysis for the MRR_m_, Ra, and TTV

The MRR_m_, Ra, and TTV results shown in Table 3 were calculated by using the range analysis and variance analysis methods; their range analysis and variance analysis results for the MRR_m_, Ra, and TTV are shown in Table 4, Table 5, Table 6, Table 7, Table 8 and Table 9, respectively. In the tables, k_MRRm_, k_Ra_, and k_TTV_ indicate the average values of the MRR_m_, Ra, and TTV, respectively, of the experimental results under the same level of each process parameter, and R_MRRm_, R_Ra_, and R_TTV_ indicate the change range of k_MRRm_, k_Ra_, and k_TTV_, respectively, which characterise the variation range of each process parameter’s experimental results within its values range; this range value is used to judge the effect order of each process parameter. The greater the R, the more significant the process parameter’s effect on the experimental results and the more critical the process parameter is. The ratio of the mean square to error for each process parameter was used to calculate F_MRRm_, F_Ra_, and F_TTV_, as well as to compare them with the relevant data in the F-value distribution table to characterise the significance of each process parameter; the larger the value, the more significant the effect. The ratio of the sum of squares to the total was used to calculate the MRR_m_ contribution (%), Ra contribution (%), and TTV contribution (%), which were then used to characterise the contribution of each process parameter to the MRR_m_, Ra, and TTV.

From the values of the R_MRRm_, F_MRRm_, and MRR_m_ contribution (%) shown in Table 4 and Table 5, it can be seen that the primary and secondary order of the effects of each process parameter on MRR_m_ was as follows: lapping plate type (A) > abrasive size (C) > binary interaction between lapping plate type and abrasive size (A × C) > binary interaction between lapping plate type and lapping normal-pressure (A × B) > abrasive concentration (D) > binary interaction between lapping normal-pressure and abrasive size (B × C) > lapping normal-pressure (B); among them: the A, C, and (A × C) process parameters were found to have the most significant effects on MRR_m_, with contributions of 36.14%, 33.50%, and 16.36%, respectively. These values indicate that the lapping plate type and abrasive size should be considered and then reasonably selected in the lapping process of SiC.

It can be seen from the R_Ra_, F_Ra_, and Ra contribution (%) values from Table 6 and Table 7 that the primary and secondary order of the effects of each process parameter on Ra was as follows: C > A > (A × C) > B > (B × C) > (A × B) > D; among them: the C, A, and (A × C) process parameters also had the most significant effects on Ra, with total contributions as high as 91.61% (35.44%, 32.53%, and 23.64%, respectively), which was similar to the effects on those of MRR_m_.

It can be seen in the R_TTV_, F_TTV_, and TTV contribution (%) values from Table 8 and Table 9 that the process parameters on TTV can be arranged in descending order as follows: (A × C) > (A × B)> (B × C) > B > C > A > D; among them: the (A × C), (A × B), (B × C), and B process parameters had the most significant effects on TTV, and their total contributions were as high as 88.59%. Furthermore, it is worth noting that the contribution of (A × C) alone to TTV was as high as 51.07%. This shows that in the lapping process of SiC, if we want to pursue a better TTV, we should pay special attention to the binary interaction effect between the lapping plate type and the abrasive size in order to reasonably select them.

### 3.2. Effect Curve Analysis

**Figure 5:**
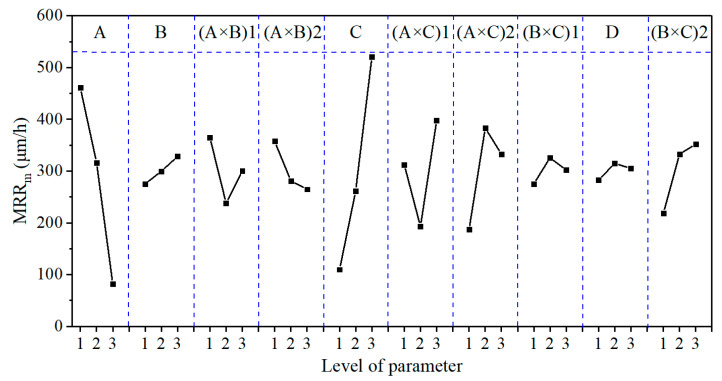
The effect plot for MRR_m_.

**Figure 6:**
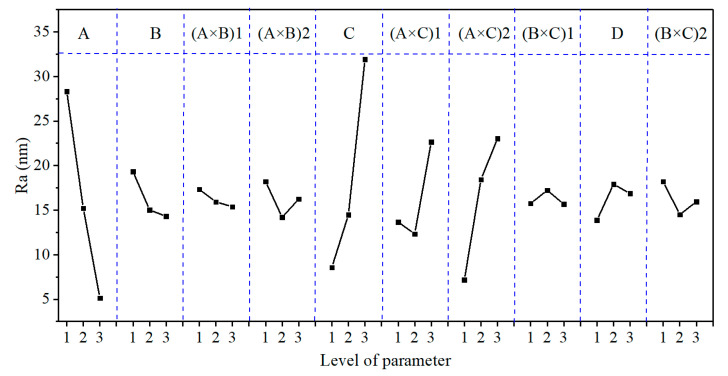
The effect plot for Ra.

**Figure 7:**
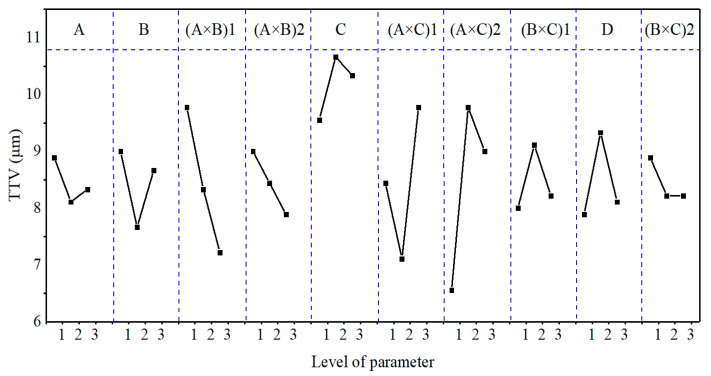
The effect plot for TTV.

Figure 5, Figure 6 and Figure 7 show the effect curves of MRR_m_, Ra, and TTV with each process parameter, respectively. From these figures, it can be seen that different lapping plates (A) exhibited different processing performance levels. When using the cast iron plate (with a hardness of 175 HV), the MRR_m_, Ra, and TTV were the largest; when using the polyurethane pad (with a hardness of 35 HD), the MRR_m_ and Ra were the smallest; when using the copper plate (with a hardness of 50 HV), the TTV was the smallest. It can also be seen that as the lapping normal-pressure (B) increased, MRR_m_ continually increased, Ra continually decreased, and TTV first decreased and then increased. Additionally, when the abrasive size (C) increased, MRR_m_ increased, Ra continually decreased, and TTV first decreased and then increased. Furthermore, the MRR_m_, Ra, and TTV increased and then decrease as the abrasive concentration (D) increased. It was also found that the three sets of binary interaction effects of (A × B), (A × C), and (B × C) showed significant differences in the effects on MRR_m_, Ra, and TTV. MRR_m_ and Ra decreased and then increased with the interaction of (A × B), and TTV continually decreased; MRR_m_, Ra, and TTV continually increased with the interaction of (A × C); MRR_m_, Ra, and TTV increased and then decreased with the interaction of (B × C). Finally, the MRR_m_ was the highest under the parameter condition of A1B3C3D2 (Exp. No. 9). When the lapping plate, lapping normal-pressure, abrasive size, and abrasive concentration were a cast-iron plate, 15 kg, 5 μm, and 2.0 wt.%, respectively, the processing efficiency was the highest. Under the parameter condition of A3B3C1D1, i.e., the lapping plate, lapping normal-pressure, abrasive size, and abrasive concentration were a polyurethane plate, 15 kg, 1 μm, and 0.5 wt.%, respectively, the Ra value was the smallest; that is, the best-machined substrate surface could be obtained. When the conditions of the A2B2C1D1, i.e., the abrasive plate, lapping normal-pressure, abrasive size, and abrasive concentration were a copper plate, 10 kg, 1 μm, and 0.5 wt.%, respectively, the TTV value was the smallest; that is, the substrate surface had the best flatness after lapping.

The main reasons for these results are as follows. First, the lapping plate type (A) affected the contact state between the SiC substrate, the abrasive, and the lapping plate, and it also affected the abrasive movement form during the lapping process, thus influencing MRR_m_, Ra, and TTV. From Figure 4, Equations (2), (3), and (6), it can be seen that the greater the lapping plate hardness, the smaller the abrasive embedded depth in the lapping plate and the greater the relative depth of the abrasive pressed into the substrate. Moreover, this also led to stronger effects of abrasive rolling, scratching, and micro-cutting between the lapping plate and the substrate during the relative movement of the lapping plate and the substrate, so the cast iron plate was able to achieve a higher substrate MRR_m_; however, due to the small total depth of the abrasive embedded in the cast iron disc and the substrate, only the relatively large particle size abrasive was involved in effective lapping and the density of the abrasive trajectory through the substrate surface was low, so the obtained Ra was higher and some of the larger abrasives left micro-cutting scratches on the substrate surface, resulting in a higher TTV. The abrasive had a significant embedding depth on the less hard copper disc but the depth of the abrasive pressed into the substrate was relatively small, so the MRR_m_ was relatively small. In contrast, the plasticity of the copper plate was better than the cast iron plate, so the depth of the abrasive embedding into the substrate was not sufficient to produce brittle cracks under the action of yielding. Hence, the processed substrate Ra was low. At the same time, the relatively large abrasive was more profoundly embedded into the copper plate, thus allowing the smaller abrasives to participate in the lapping under the action of lapping normal-pressure and resulting in a denser trajectory across the substrate surface in the same amount of time and a wafer with a low TTV. The polyurethane plate had the lowest hardness, and the abrasive was embedded in the pad to the greatest depth, though the abrasive was pressed into the substrate to the smallest depth, so the MRR_m_ was the smallest. At the same time, the polyurethane pad easily allowed the abrasive to be fully embedded, so the surface roughness, Ra, of the processed wafer was the lowest and the TTV was low.

Second, the lapping normal-pressure (B) mainly affected the force and its motion state between the abrasive, the SiC substrate, and the lapping plate. As the lapping normal-pressure increased, the force between the abrasive, the substrate, and the lapping plate increased and the depth of the single abrasive embedded in the lapping plate’s surface gradually increased. The abrasive gradually changed from three-body friction to two-body friction motion during the lapping process’s relative movement. As the substrate removal gradually changed from rolling, scratching, and abrasive breaking to ploughing and micro-cutting, the micro-cutting depth gradually increased and the effective abrasive involved in lapping increased. Therefore, the MRR_m_ increased as the lapping normal-pressure increased. However, due to the increase in the force between the substrate, the abrasive, and the lapping plate, the force on the abrasive also increased, resulting in more scratches on the substrate surface and consequentially an increasing Ra.

Third, the abrasive size (C) mainly affected the number of abrasives involved in the lapping process and the force of a single abrasive particle, thereby affecting MRR_m_, Ra, and TTV. Since the hardness of the lapping plates were all lower than that of the SiC substrate, the diamond abrasives were easily embedded on the lapping plate’s surface, which hindered the tumbling movement of the abrasive and caused two-body friction; the material was mainly removed by micro-cutting during the lapping process. As the abrasive size increased, the micro-cutting effect and, accordingly, the MRR_m_ increased; however, following the relationship between the number of abrasives in the slurry and the abrasive size shown in Equation (8) (where M_L_ is the total mass of the slurry and *w*_a_ is the abrasive concentration), it can be seen that when the abrasive size increased, the volume of the single abrasive increased and the total number of abrasive in the lapping slurry decreased. Simultaneously, the large abrasive particles were held on the lapping plate’s surface to produce two-body friction movement on the substrate. Increases in the material removal depth resulted in reductions in the lapped surface’s quality and increases in the Ra.

(8) $$N=\frac{6M_{L}w_{a}}{\rho_{a}\pi d_{a}{}^{3}}$$

Fourth, abrasive concentration (D) affected the number of effective abrasives in the lapping process, as well as the MRR_m_, Ra, and TTV. It can be seen from Equations (5) and (6) that increases in the concentration of the abrasive led to the number of abrasives in the slurry increasing and more abrasives participating in the lapping process, which then increased the MRR_m_. However, when the abrasive concentration was too high, the abrasives easily accumulated, the abrasive’s unevenness increased, the number of effective abrasives reduced, and the abrasive’s micro-cutting effect weakened. Therefore, as the abrasive concentration increased, the MRR_m_ first increased and then decreased and the Ra and TTV first increased and then decreased.

Based on the above analysis, it can be seen that the effect significance levels of the lapping plate type (A), lapping normal-pressure (B), abrasive size (C), and abrasive concentration (D), as well as their interactions, on the three evaluation indexes (MRR_m_, Ra, and TTV) were different. After analysing the effect curve, the optimal process parameter combinations obtained under each of the evaluation indexes were not quite the same, so it was not easy to choose a suitable optimal combination. Additionally, the two experimental combinations of A3B3C1D1 (the smallest surface roughness, Ra) and A2B2C1D1 (the smallest TTV value and the best flatness) were not included in this orthogonal experiment, which cannot be compared to the generally chosen experimental combinations. Therefore, to obtain a comprehensive optimal process parameter combination that can meet each evaluation index, other methods are still required for optimisation analysis.

### 3.3. Grey Relational Analysis

Grey relational analysis is a method suitable for determining the relational grade between multiple process parameters and multiple experimental evaluation indexes, and it has significant ability to solve multi-evaluation-index optimisation problems. The single-crystal SiC lapping process is a grey system with incomplete information formed by the interaction between various process parameters and various evaluation indexes, and it is suitable for use with grey relational analysis. Grey relational analysis mainly includes the following main steps:

(1) Dimensionless normalisation processing is performed on the original data obtained through experiments. Since each evaluation index has significant differences in its meaning, value standard, and levels, their data dimensions are not quite the same. Generally, they cannot be used for direct comparison, it is necessary to carry out the dimensionless normalisation of the evaluation index data to make the data of each evaluation index comparable. In this experiment, the larger the MRR_m_, the better, so the original data were processed by the large-the-better characteristic data processing formula shown in Equation (9); meanwhile, the Ra and TTV were required to be as small as possible, so the original data were processed by the smaller-the-better characteristic data processing formula shown in Equation (10) [15].

(9) $$x_{ij}=\frac{y_{ij}-\min_{j}y_{ij}}{\max_{j}y_{ij}-\min_{j}y_{ij}}(i=1,\cdots \cdots m;\;j=1,\cdots \cdots n)$$

(10) $$x_{ij}=\frac{\max_{j}y_{ij}{-y}_{ij}}{\max_{j}y_{ij}-\min_{j}y_{ij}}(i=1,\cdots \cdots m;\;j=1,\cdots \cdots n)$$

where *x_ij_* is the normalised data (ND), *y_ij_* is the original experimental data obtained through the experiment, *m* is the number of experiments (taking *m* = 27), and *n* is the number of experimental levels (taking *n* = 3).

(2) The grey relational coefficient (GRC) can be calculated by Equation (11) [17].

(11) $$\gamma \left(x_{0j},x_{ij}\right)=\frac{\Delta_{\min}+\xi \Delta_{\max}}{\Delta_{ij}+\xi \Delta_{\max}};\;0<\gamma \left(x_{0j},x_{ij}\right)\leq 1\;(i=1,\cdots \cdots m;\;j=1,\cdots \cdots n)$$

where *γ*(*x*_0*i*_, *x_ij_*) is the grey relational coefficient; ζ is the resolution coefficient (generally taken as 0.5); and Δ*_ij_*, Δ_min_, and Δ_max_ are the absolute value of the difference between *x*_0*j*_ and *x_ij_*, the minimum value of Δ*_ij_*, and the maximum value of Δ*_ij_*, respectively.

(3) GRG is the weighted sum of the grey relational coefficients. Different weights indicate different degrees of importance to the evaluation index, so it is necessary to calculate each evaluation index’s weight. The entropy method is an objective weighting method. If the information entropy of a specific evaluation index is smaller in the actual measurement process, the difference between this evaluation index’s experimental results is more significant. The greater the effect in the comprehensive evaluation, the greater the corresponding evaluation index’s weight. According to the GRC calculated in Equation (11), the entropy method can be used to calculate each evaluation index’s weight value, and the weight *P_ij_* of the *j*-th evaluation index in the *i*-th experiment group can be calculated by Equation (12).

(12) $$p_{{}_{ij}}=\frac{\gamma \left(x_{0j},x_{ij}\right)}{\sum_{i=1}^{m}\gamma \left(x_{0j},x_{ij}\right)}$$

The matrix formed by the weight *P_ij_* is denoted as P. Using this matrix, the information entropy value *H_j_,* information redundancy *e_j_* of the *j*-th evaluation index, and the final weight *ω_j_* can be calculated accordingly. The calculation formulae are shown in Equations (13)–(15) [21], respectively. The calculated information entropy (*H_j_*), information entropy redundancy (*e_j_*), and weight (*ω_j_*) are shown in Table 10.

(13) $$H_{j}=\frac{-\sum_{i=1}^{m}\left(p_{ij}*\ln p_{ij}\right)}{\ln m}$$

(14) $$e_{j}=1{-H}_{j}$$

(15) $$\omega_{j}=\frac{e_{j}}{\sum_{j=1}^{n}e_{j}}=\frac{1{-H}_{j}}{\sum_{j=1}^{n}\left(1{-H}_{j}\right)}$$

(4) The GRG is the relational grade between the experimental process parameters and experimental evaluation indexes, and it can be calculated by Equation (16) [22]:

(16) $$\gamma \left(x_{0},x_{i}\right)=\sum_{i=1}^{n}\omega_{j}\gamma \left(x_{0j},x_{ij}\right)\;(i=1,\cdots \cdots m;\;j=1,\cdots \cdots n)$$

where *ω_j_* is the weight of each evaluation index, as calculated and determined by Equation (15).

After the above-discussed data processing, the results of the ND, GRC, GRG, and GRG rank of the orthogonal experiment are shown in Table 11. In the table, GRG is used for a performance evaluation that comprehensively considers the three evaluation indexes of MRR_m_, Ra, and TTV. The larger the GRG, the closer the corresponding experimental results were to the ideal value. The performance of any of the three evaluation indexes of MRR_m_, Ra, and TTV could be determined by the effect curve analysis results in Section 3.2. Therefore, the complex multi-evaluation-index optimisation problem could be transformed into a single GRG optimisation problem. Among the 27 groups of experiments, according to the GRG value, the best comprehensive performance was that of Exp. No. 25 (GRG = 0.72904), and the corresponding parameter combination was A3B3C1D2.

**Figure 8:**
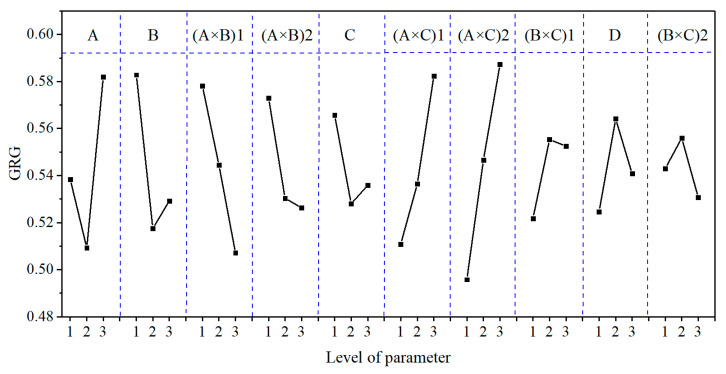
The effect plot for GRG.

The GRG values (Table 11) calculated by the grey correlation analysis were subjected to range, variance, and effect curve analyses. The results are shown in Table 12 and Table 13 and Figure 8, respectively.

From the R_GRG_ and F_GRG_ values and the GRG contribution (%) shown in Table 12 and Table 13, it can be seen that the main order of each process parameter’s effect on GRG was (A × C) > (A × B) > A > B > (B × C) > C > D. The (A × C), (A × B), A, B, and (B × C) factors had the most significant effects on GRG, with contributions to GRG of 35.61%, 20.12%, 13.93%, 12.65%, and 7.70%, respectively. This conclusion differs from that of Section 3.1, mainly because Section 3.1 only considers each process parameter’s effect on the MRR_m_, Ra, and TTV individually rather than the comprehensive performance.

As can be seen from Figure 8, the GRG value first decreased and then increased with the decrease in lapping plate hardness and the increase in lapping normal-pressure and abrasive size. The GRG value first increased and then decreased as the abrasive concentration increased, continued to decrease with the interaction effects of (A × B), continued to increase with the interaction effects of (A × C), and increased and then decreased with the interaction effects of (B × C). The optimum combination of the process parameters obtained from the effect curve analysis under multiple evaluation indexes was A3B1C1D2; that is, when the lapping plate, lapping normal-pressure, abrasive size, and abrasive concentration were a polyurethane pad, 5 kg, 1 μm, and 1.0 wt.%, respectively, we found the most significant GRG value (i.e., the best overall performance of the lapping process). However, this combination was not reflected in the abovementioned experiments.

### 3.4. Validation of the Experimental Results

**Figure 9:**
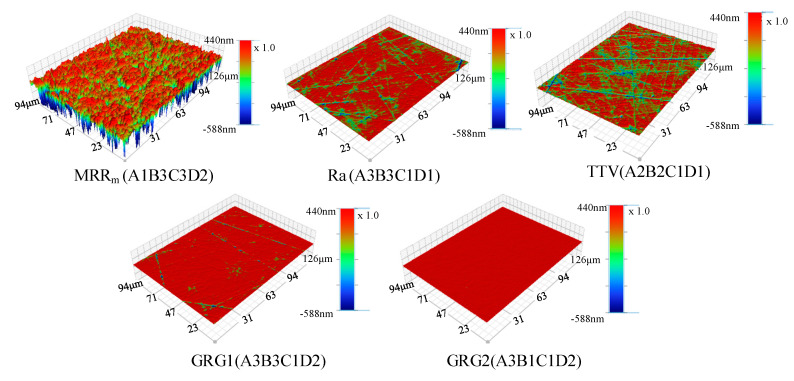
The surface topographies of the optimal combination for MRR_m_ (A1B3C3D2), Ra (A3B3C1D1), TTV (A2B2C1D1), GRG1 (A3B3C1D2), and GRG2 (A3B1C1D2).

From the above analysis, it can be seen that the optimal combinations of MRR_m_ (A1B3C3D2), Ra (A3B3C1D1), and TTV (A2B2C1D1) obtained by effect curve analysis under a single evaluation index and the optimal combinations of GRG (A3B3C1D2 and A3B1C1D2) obtained by grey relational analysis were different. To verify the grey correlation analysis’s validity, the experiments were repeated according to the optimal combination determined by the grey relational analysis and compared to the optimal combination determined by the effect curve analysis under the single evaluation index. The experimental protocol and results are shown in Table 14, and the surface morphology of the processed SiC is shown in Figure 9.

From Table 14 and Figure 9, it can be seen that after optimisation by the grey relational analysis method, the MRR_m_ decreased from 827.9 to 90.2 μm/h; however, Ra decreased from 3.642 to 0.769 nm and TTV decreased from 4 to 3 μm. Furthermore, both the number of scratches and pits were significantly reduced; in the surface topographies of optimal combination for GRG2 (A3B1C1D2), there were almost no visible scratches and pits. Additionally, GRG increased by 38.17%, 3.38%, and 4.39% as the MRR_m_, Ra, and TTV decreased, respectively; from the perspective of later polishing and application, when the MRR_m_ is high enough, it should be given more attention than the process parameters to obtain a strong surface quality. Thus, it was concluded that the process parameters obtained after optimisation using the grey relational analysis method were the best. The overall lapping performance of the SiC substrate was also the best. This shows that it is feasible and practical to use the grey relational analysis method to transform the optimisation problem of multiple evaluation indexes (MRR_m_, Ra, and TTV) into a single-evaluation-index optimisation problem regarding GRG, and it can be used to optimise the lapping process of SiC and to provide a new idea to obtain a lapping process with strong, comprehensive performance.

## 4. Conclusions

(1) The lapping process of single-crystal SiC is a process in which the parameters and their interactions are coordinated with each other. Only when the process parameters and levels are reasonably selected can an SiC with a high-efficiency and high-quality flattening process be realised. Range and variance analyses have shown that the three factors of lapping plate type (A), abrasive size (C), and the binary interaction effect between lapping plate type and abrasive size (A × C) have the most significant effects on MRR_m_ and Ra. The contribution of these three factors to MRR_m_ and Ra is as high as 85%. The binary interaction effect between lapping plate type and abrasive size (A × C) has the most significant effect on TTV, with a contribution rate of up to 51.07%. Altogether, these results show that in the lapping process of SiC, the lapping plate type and abrasive size should be selected under reasonable consideration.

(2) The lapping plate type and lapping normal-pressure affect the contact states among the SiC substrate, the abrasive, and the lapping plate; the force and movement of the abrasive; and the form of the abrasive inlay in the lapping plate. The abrasive size and concentration affect the number of effective abrasives involved in the lapping process and the force on a single abrasive. The effect curve analysis showed that the substrate’s MRR_m_ gradually increased as the lapping plate hardness and abrasive size increased; the Ra after lapping also increased. As the lapping normal-pressure increased, it was found that the MRR_m_ increased, the Ra decreased, and the TTV first decreased and then increased. As the abrasive concentration increased, the MRR_m_, Ra, and TTV increased and then decreased.

(3) To obtain SiC lapping process parameters with strong, comprehensive lapping performance (i.e., high MRR_m_, small Ra, and low TTV), the multi-evaluation-index optimisation problem was transformed into a single-evaluation-index optimisation problem regarding the GRG using grey relational analysis. The results showed that the binary interaction effects between lapping plate type and abrasive size (A × C), lapping plate type and lapping normal-pressure (A × B), and lapping plate type and lapping normal-pressure are the parameters that have the most significant effects on the lapping process of SiC. A smooth surface with an MRR_m_ of 90.2 μm/h, an Ra of 0.769 nm, and a TTV of 3 μm was obtained under optimal process conditions using the grey relational analysis, which was better than the overall performance of the machined surface obtained by the intuitive analysis under the single evaluation index.

## Figures and Tables

**Table 1:** Machining parameters and their levels.

Symbol	Machining Parameters	Unit	Level 1	Level 2	Level 3
A	Lapping plate types		Cast iron plate	Copper plate	Polyurethane pad
B	Lapping normal-pressure	Kg	5.0	10.0	15.0
C	Abrasive size	μm	1.0	3.0	5.0
D	Abrasive concentration	wt.%	0.5	1.0	2.0

**Table 2:** Header design of the L_27_(3^13^) orthogonal array.

Col. No.	1	2	3	4	5	6	7	8	9	10	11	12	13
**Symbol**	A	B	(A × B)1	(A × B)2	C	(A × C)1	(A × C)2	(B × C)1			(B × C)2	D	

**Table 3:** Experimental results for MRR_m_, Ra, and TTV using the L_27_(3^13^) orthogonal experiment.

Exp. No. (Combination)	MRR_m_ (μm/h)	Ra (nm)	TTV (μm)	Exp. No. (Combination)	MRR_m_ (μm/h)	Ra (nm)	TTV (μm)	Exp. No. (Combination)	MRR_m_ (μm/h)	Ra (nm)	TTV (μm)
1(A1B1C1D1)	109.0	2.605	9	10(A2B1C1D2)	29.1	12.482	6	19(A3B1C1D3)	66.0	4.619	8
2(A1B1C2D2)	635.2	38.374	13	11(A2B1C2D3)	212.0	10.736	10	20(A3B1C2D1)	63.7	5.364	10
3(A1B1C3D3)	730.5	62.644	12	12(A2B1C3D1)	405.8	30.966	8	21(A3B1C3D2)	48.5	6.153	5
4(A1B2C1D2)	222.1	8.713	6	13(A2B2C1D3)	72.8	8.223	6	22(A3B2C1D1)	53.5	3.335	11
5(A1B2C2D3)	353.4	12.044	8	14(A2B2C2D1)	243.3	6.950	5	23(A3B2C2D2)	74.2	5.779	9
6(A1B2C3D1)	612.8	53.684	10	15(A2B2C3D2)	722.0	30.380	9	24(A3B2C3D3)	121.5	6.120	5
7(A1B3C1D3)	311.8	9.019	5	16(A2B3C1D1)	53.5	3.929	9	25(A3B3C1D2)	98.3	3.552	5
8(A1B3C2D1)	347.1	17.892	7	17(A2B3C2D2)	337.3	8.482	9	26(A3B3C2D3)	132.1	5.463	9
9(A1B3C3D2)	827.9	49.889	10	18(A2B3C3D3)	769.2	24.784	11	27(A3B3C3D1)	76.2	5.756	5

**Table 4:** Range analysis for MRR_m_.

	A	B	(A × B)1	(A × B)2	C	(A × C)1	(A × C)2	(B × C)1	D	(B × C)2
**k_MRRm 1_**	461.1	275.1	320.4	313.3	109.6	312.2	186.9	275.2	283.2	218.3
**k_MRRm 2_**	316.1	255.5	238.0	280.7	261.0	193.0	383.4	325.8	314.5	332.7
**k_MRRm 3_**	81.6	328.2	300.3	264.8	476.5	353.5	288.5	257.7	261.1	307.7
**R_MRRm_**	379.5	72.6	82.5	48.5	366.9	160.5	196.5	68.1	53.5	114.4

**Table 5:** ANOVA analysis for MRR_m_.

Source	Sum of Squares	Degree of Freedom	Mean Square	F_MRRm_ Value	Critical Value of F	Significant Levels	Contribution (MRR_m_, %)
**A**	660,170.84041	2	330,085.42020	23.20348	F_0_._1_(2,6) = 3.46 *	***	36.14%
**B**	25,420.06381	2	12,710.03191	0.89346	F_0_._05_(2,6) = 5.14 **		1.39%
**C**	611,977.54689	2	305,988.77344	21.50959	F_0_._01_(2,6) = 10.925 ***	***	33.50%
**D**	65,102.29047	2	32,551.14523	2.28819			3.56%
**(A × B)**	44,277.10212	4	11,069.27553	0.77812	F_0_._1_(4,6) = 3.29 *		2.42%
**(A × C)**	298,853.25020	4	74,713.31255	5.25200	F_0_._05_(4,6) = 4.53 **	**	16.36%
**(B × C)**	35,529.12349	4	8882.28087	0.62438	F_0_._01_(4,6) = 9.148 ***		1.95%
**Error**	85,354.12576	6	14,225.68763				4.67%
**Total**	1,826,684.34314	26					100.00%

**Table 6:** Range analysis for Ra.

	A	B	(A × B)1	(A × B)2	C	(A × C)1	(A × C)2	(B × C)1	D	(B × C)2
**k_Ra 1_**	28.318	19.327	17.339	18.216	8.571	13.675	7.170	15.747	13.879	18.200
**k_Ra 2_**	15.215	15.025	15.933	14.197	14.478	12.330	18.438	17.242	17.906	14.498
**k_Ra 3_**	5.127	14.307	15.388	16.246	31.914	22.654	23.051	15.671	16.875	15.961
**R_Ra_**	23.191	5.019	1.951	4.020	23.342	10.324	15.880	1.571	4.028	3.702

**Table 7:** ANOVA analysis for Ra.

Source	Sum of Squares	Degree of Freedom	Mean Square	F_Ra_ Value	Critical Value of F	Significant Levels	Contribution (Ra, %)
**A**	2433.86682	2	1216.93341	29.36944	F_0_._1_(2,6) = 3.46 *	***	32.53%
**B**	132.64259	2	66.32130	1.60060	F_0_._05_(2,6) = 5.14 **		1.77%
**C**	2651.25670	2	1325.62835	31.99268	F_0_._01_(2,6) = 10.925 ***	***	35.44%
**D**	62.58883	2	31.29441	0.75526			0.84%
**(A × B)**	90.96419	4	22.74105	0.54883	F_0_._1_(4,6) = 3.29 *		1.22%
**(A × C)**	1768.37954	4	442.09489	10.66951	F_0_._05_(4,6) = 4.53 **	***	23.64%
**(B × C)**	92.90940	4	23.22735	0.56057	F_0_._01_(4,6) = 9.148 ***		1.24%
**Error**	248.61215	6	41.43536				3.32%
**Total**	7481.22022	26					100.00%

**Table 8:** Range analysis for TTV.

	A	B	(A × B)1	(A × B)2	C	(A × C)1	(A × C)2	(B × C)1	D	(B × C)2
**K_TTV 1_**	8.89	9.00	9.78	9.00	9.56	8.44	6.56	8.00	7.89	8.89
**K_TTV 2_**	8.11	7.67	8.33	8.44	10.67	7.11	9.78	9.11	9.33	8.22
**K_TTV 3_**	8.33	8.67	7.22	7.89	10.33	9.78	9.00	8.22	8.11	8.22
**R_TTV_**	0.78	1.33	2.56	1.11	1.11	2.67	3.22	1.11	1.44	0.67

**Table 9:** ANOVA analysis for TTV.

Source	Sum of Squares	Degree of Freedom	Mean Square	F_TTV_ Value	Critical Value of F	Significant Levels	Contribution (TTV, %)
**A**	2.88889	2	1.44444	1.21875	F_0_._1_(2,6) = 3.46 *		1.78%
**B**	8.66667	2	4.33333	3.65625	F_0_._05_(2,6) = 5.14 **	*	5.34%
**C**	5.85185	2	2.92593	2.46875	F_0_._01_(2,6) = 10.925 ***		3.61%
**D**	2.66667	2	1.33333	1.12500			1.64%
**(A × B)**	35.11111	4	8.77778	7.40625	F_0_._1_(4,6) = 3.29 *	**	21.63%
**(A × C)**	82.88889	4	20.72222	17.48438	F_0_._05_(4,6) = 4.53 **	***	51.07%
**(B × C)**	17.11111	4	4.27778	3.60937	F_0_._01_(4,6) = 9.148 ***	*	10.54%
**Error**	7.11111	6	1.18519				4.38%
**Total**	162.29630	26					100.00%

**Table 10:** Calculation results of the entropy method.

Evaluation Index	MRR_m_ (*j =* 1)	Ra (*j =* 2)	TTV (*j =* 3)
***H_j_***	0.98355	0.98330	0.98330
***e_j_***	0.01645	0.01670	0.01670
***ω_j_***	0.32994	0.33503	0.33503

**Table 11:** The ND, GRC, GRG, and order of GRG for the orthogonal results.

Exp. No.	MRR_m_ (*j =* 1)		Ra (*j =* 2)		TTV (*j =* 3)		GRG	Rank
	ND	GRC	ND	GRC	ND	GRC		
1	0.10007	0.83323	1.00000	0.33333	0.50000	0.50000	0.55410	11
2	0.75878	0.39721	0.40424	0.55295	0.00000	1.00000	0.65131	3
3	0.87805	0.36283	0.00000	1.00000	0.12500	0.80000	0.72278	2
4	0.24156	0.67425	0.89827	0.35758	0.87500	0.36364	0.46409	22
5	0.40598	0.55189	0.84278	0.37236	0.62500	0.44444	0.45574	23
6	0.73077	0.40625	0.14924	0.77013	0.37500	0.57143	0.58351	7
7	0.35395	0.58551	0.89317	0.35889	1.00000	0.33333	0.42510	27
8	0.39806	0.55676	0.74538	0.40148	0.75000	0.40000	0.45222	24
9	1.00000	0.33333	0.21244	0.70181	0.37500	0.57143	0.53656	16
10	0.00000	1.00000	0.83549	0.37439	0.87500	0.36364	0.57721	9
11	0.22896	0.68591	0.86457	0.36642	0.37500	0.57143	0.54050	14
12	0.47162	0.51461	0.52762	0.48656	0.62500	0.44444	0.48171	20
13	0.05467	0.90143	0.90643	0.35551	0.87500	0.36364	0.53836	15
14	0.26818	0.65089	0.92763	0.35023	1.00000	0.33333	0.44377	26
15	0.86743	0.36565	0.53738	0.48199	0.50000	0.50000	0.44964	25
16	0.03060	0.94233	0.97794	0.33831	0.50000	0.50000	0.59176	6
17	0.38580	0.56446	0.90211	0.35660	0.50000	0.50000	0.47322	21
18	0.92659	0.35049	0.63058	0.44225	0.25000	0.66667	0.48714	19
19	0.04625	0.91533	0.96646	0.34096	0.62500	0.44444	0.56513	10
20	0.04331	0.92029	0.95404	0.34387	0.37500	0.57143	0.61028	5
21	0.02432	0.95361	0.94090	0.34700	1.00000	0.33333	0.54257	13
22	0.03050	0.94251	0.98784	0.33606	0.25000	0.66667	0.64689	4
23	0.05647	0.89853	0.94713	0.34551	0.50000	0.50000	0.57972	8
24	0.11571	0.81207	0.94146	0.34687	1.00000	0.33333	0.49582	18
25	0.08667	0.85227	0.98422	0.33688	0.00000	1.00000	0.72904	1
26	0.12893	0.79500	0.95239	0.34426	0.50000	0.50000	0.54514	12
27	0.05896	0.89452	0.94751	0.34542	1.00000	0.33333	0.52254	17

**Table 12:** Range analysis for GRG.

	A	B	(A × B)1	(A × B)2	C	(A × C)1	(A × C)2	(B × C)1	D	(B × C)2
**K_GRG 1_**	0.53838	0.58284	0.57808	0.57296	0.56574	0.51077	0.49575	0.52178	0.52466	0.54298
**K_GRG 2_**	0.50926	0.51751	0.54439	0.53038	0.52799	0.53639	0.54653	0.55530	0.56411	0.55593
**K_GRG 3_**	0.58191	0.52919	0.50707	0.52619	0.53581	0.58238	0.58726	0.55247	0.54078	0.53064
**R_GRG_**	0.07265	0.06534	0.07101	0.04677	0.03775	0.07161	0.09151	0.03352	0.03945	0.02529

**Table 13:** ANOVA analysis for GRG.

Source	Sum of Squares	Degree of Freedom	Mean Square	F_GRG_ Value	Critical Value of F	Significant Levels	Contribution (GRG, %)
**A**	0.02406	2	0.01203	9.97098	F_0.1_(2,6) = 3.46 *	**	13.93%
**B**	0.02185	2	0.01093	9.05469	F_0.05_(2,6) = 5.14 **	**	12.65%
**C**	0.00715	2	0.00357	2.96182	F_0.01_(2,6) = 10.925 ***		4.14%
**D**	0.00288	2	0.00144	1.19311			1.67%
**(A × B)**	0.03477	4	0.00869	7.20332	F_0.1_(4,6) = 3.29 *	**	20.12%
**(A × C)**	0.06153	4	0.01538	12.74926	F_0.05_(4,6) = 4.53 **	***	35.61%
**(B × C)**	0.01330	4	0.00333	2.75603	F_0.01_(4,6) = 9.148 ***		7.70%
**Error**	0.00724	6	0.00121				4.19%
**Total**	0.17278	26					100.00%

**Table 14:** Experimental protocol and results of the confirmation experiment.

Index (Combination)	Machining Parameters			Experimental Results				
	Lapping Plate	Normal Pressure (kg)	Abrasive Size (μm)	Abrasive Concentration (wt.%)	MRR_m_ (μm/h)	Ra (nm)	TTV (μm)	GRG
**MRR_m_ (A1B3C3D2)**	Cast iron	15.0	5.0	1.0	827.9	50.762	10	0.53656
**Ra (A3B3C1D1)**	Polyurethane	15.0	1.0	0.5	111.1	3.642	5	0.71710
**TTV (A2B2C1D1)**	Copper	10.0	1.0	0.5	92.7	5.071	4	0.71015
**GRG1 (A3B3C1D2)**	Polyurethane	15.0	1.0	1.0	98.3	3.381	4	0.72914
**GRG2 (A3B1C1D2)**	Polyurethane	5.0	1.0	1.0	90.2	0.769	3	0.74136
